# Clinical Course and Diagnostic Findings of Biopsy Controlled Presumed Immune-Mediated Polyneuropathy in 70 European Cats

**DOI:** 10.3389/fvets.2022.875657

**Published:** 2022-05-17

**Authors:** Jana van Renen, Andrea Fischer, Ninja Kolb, Franziska Wielaender, Yury Zablotski, Jasmin Nessler, Andrea Tipold, Rodolfo Cappello, Thomas Flegel, Shenja Loderstedt, Kirsten Gnirs, Kai Rentmeister, Stephan Rupp, Thilo von Klopmann, Frank Steffen, Konrad Jurina, Omar V. Del Vecchio, Martin Deutschland, Florian König, Gualtiero Gandini, Tom Harcourt-Brown, Marion Kornberg, Ezio Bianchi, Teresa Gagliardo, Marika Menchetti, Henning Schenk, Joana Tabanez, Kaspar Matiasek, Marco Rosati

**Affiliations:** ^1^Neurology Service, Centre for Clinical Veterinary Medicine, Ludwig-Maximilians Universität München, Munich, Germany; ^2^Section of Clinical and Comparative Neuropathology, Centre for Clinical Veterinary Medicine, Ludwig-Maximilians Universität München, Munich, Germany; ^3^Clinic for Ruminants With Ambulatory and Herd Health Services, Ludwig-Maximilians Universität München, Munich, Germany; ^4^Department of Small Animal Medicine and Surgery, University of Veterinary Medicine Hannover, Hannover, Germany; ^5^North Downs Specialist Referrals, The Brewerstreet Dairy Business Park, Bletchingley, United Kingdom; ^6^Small Animal Department, University of Leipzig, Leipzig, Germany; ^7^Section of Neurology and Neurosurgery, Advetia Clinic for Small Animal Medicine, Paris, France; ^8^Specialty Practice for Veterinary Neurology and Neurosurgery, Dettelbach, Germany; ^9^Tierklinik Hofheim, Hofheim, Germany; ^10^Neurology Service, Department of Small Animals, Vetsuisse Faculty, University of Zurich, Zurich, Switzerland; ^11^AniCura Tierklinik Haar, Haar, Germany; ^12^Centro Veterinario Caleidos, Albisola Superiore, Italy; ^13^Neurological Referral Service, Berlin, Germany; ^14^Fachtierarzt für Kleintiere, Wiesbaden, Germany; ^15^Department of Veterinary Medical Sciences, University of Bologna, Ozzano dell'Emilia, Italy; ^16^Langford Veterinary Services, School of Veterinary Sciences, University of Bristol, Lower Langford, United Kingdom; ^17^AniCura Tierklinik Trier, Trier, Germany; ^18^Department of Veterinary Science, University of Parma, Parma, Italy; ^19^Palermovet, Veterinary Diagnostic Center, Palermo, Italy; ^20^Neurology and Neurosurgery Division, San Marco Veterinary Clinic, Veggiano, Italy; ^21^Tierklinik Lüneburg, Lüneburg, Germany; ^22^Neurology Section, Fitzpatrick Referrals, Godalming, United Kingdom

**Keywords:** feline, neuromuscular, weakness, tetraparesis, electrodiagnostic, CIDP, GBS

## Abstract

There is a paucity of information on the clinical course and outcome of young cats with polyneuropathy. The aim of the study was to describe the clinical features, diagnostic investigations, and outcome of a large cohort of cats with inflammatory polyneuropathy from several European countries. Seventy cats with inflammatory infiltrates in intramuscular nerves and/or peripheral nerve biopsies were retrospectively included. Information from medical records and follow up were acquired via questionnaires filled by veterinary neurologists who had submitted muscle and nerve biopsies (2011–2019). Median age at onset was 10 months (range: 4–120 months). The most common breed was British short hair (25.7%), followed by Domestic short hair (24.3%), Bengal cat (11.4%), Maine Coon (8.6%) and Persian cat (5.7%), and 14 other breeds. Male cats were predominantly affected (64.3%). Clinical signs were weakness (98.6%) and tetraparesis (75.7%) in association with decreased withdrawal reflexes (83.6%) and, less commonly, cranial nerve signs (17.1%), spinal pain/hyperesthesia (12.9%), and micturition/defecation problems (14.3%). Onset was sudden (30.1%) or insidious (69.1%), and an initial progressive phase was reported in 74.3%. Characteristic findings on electrodiagnostic examination were presence of generalized spontaneous electric muscle activity (89.6%), decreased motor nerve conduction velocity (52.3%), abnormal F-wave studies (72.4%), pattern of temporal dispersion (26.1%) and unremarkable sensory tests. The clinical course was mainly described as remittent (49.2%) or remittent-relapsing (34.9%), while stagnation, progressive course or waxing and waning were less frequently reported. Relapses were common and occurred in 35.7% of the cats' population. An overall favorable outcome was reported in 79.4% of patients. In conclusion, young age at the time of diagnosis and sudden onset of clinical signs were significantly associated with recovery (*p* < 0.05). Clinical and electrodiagnostic features and the remittent-relapsing clinical course resembles juvenile chronic inflammatory demyelinating polyneuropathy (CIDP), as seen in human (children/adolescents), in many aspects.

## Introduction

Weakness is a relatively common neurological presentation in cats and besides diseases of the muscles and the neuromuscular junction, polyneuropathies (PN) are one of the leading causes for this clinical presentation (1–3). Feline PN can be classified as inherited and acquired PN of defined or unknown cause (1–5). Examples of genetic or suspected genetic PN are sphingomyelinase-deficiency PN in Siamese cats (1, 6), primary hyperchylomicronemia in different breeds (1, 7), axonal PN in Snowshoe cats (8), or distal PN in Birman cats (1, 9). Acquired PN are described to be either of metabolic (10–12), vascular (13, 14), toxic (15–21), paraneoplastic (22–24), infectious (25–28), nutritional (4, 29), or are thought to represent immune-mediated or idiopathic PN (5, 30–34). Reports of immune-mediated/idiopathic PN in cats have been published in recent years (5, 31–33, 35). Many cats made a full recovery but relapses, a chronic disease course, or rare fatalities were also reported (5, 31–33, 35–38). In clinical neurological practice, a presumptive diagnosis is frequently based on neuroanatomical localization, age of onset, and exclusion of other diseases with further confirmation obtained by electrodiagnostic studies or muscle/nerve biopsies (1–3, 6, 31, 32, 39). Little is known about PN in young cats and detailed descriptions about clinical presentation and disease course are restricted to small patients' cohorts. Thus, uncertainties regarding outcome, recovery time, possible relapses, and efficacy of treatment modalities which are necessary for adequate counseling of cat owners, remain (5, 30, 31, 40). Therefore, the aim of this study was to describe the clinical features and disease course in a large European cohort of cats with histologically confirmed inflammatory PN.

## Materials and Methods

**Figure 1:**
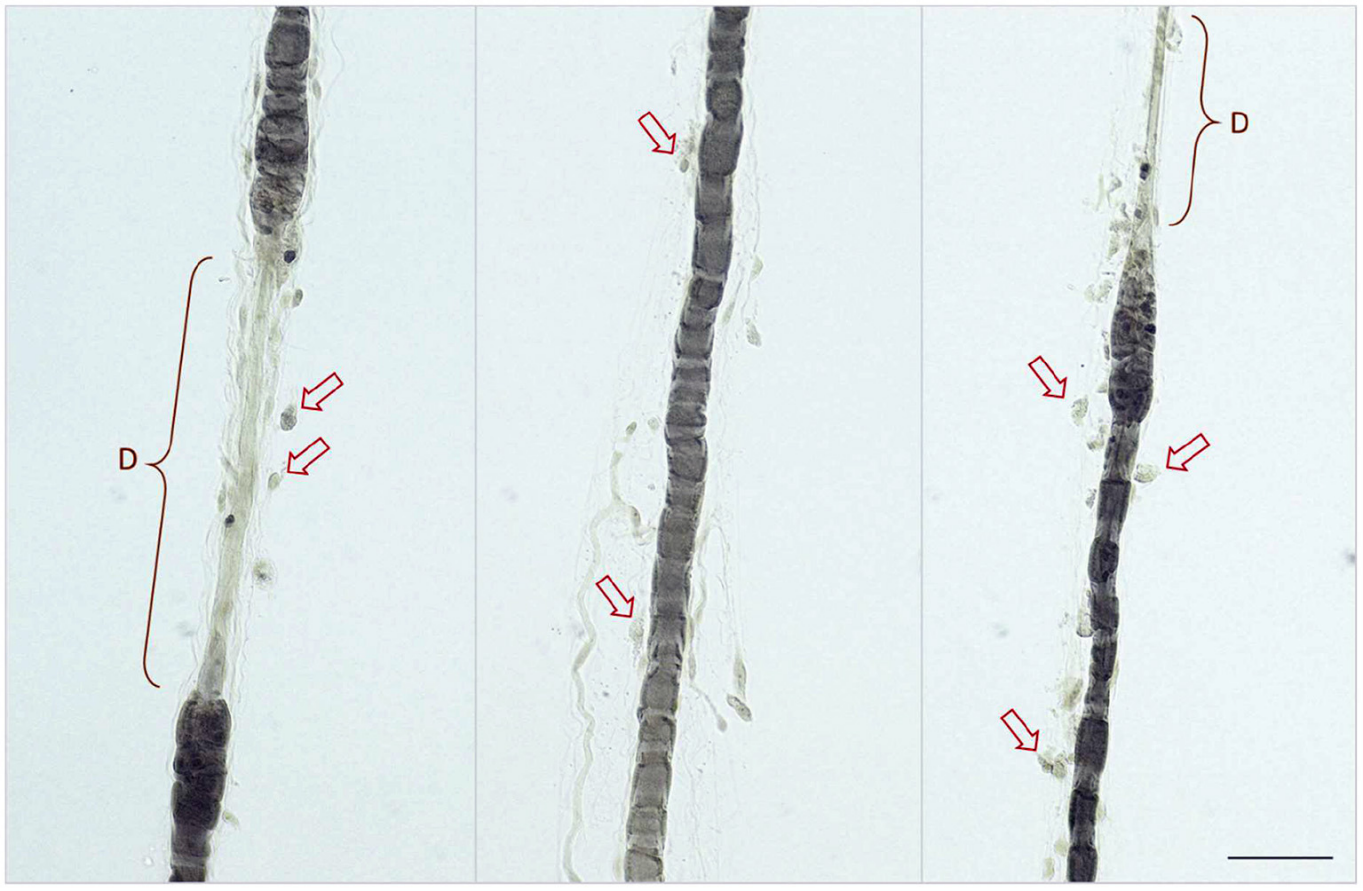
Nerve fiber teasing preparations showing multiple fiber adhesive and invasive round mononuclear cells (lymphocytes and macrophages) indicated by red arrows at the Schmidt Lanterman clefts, paranodium and along demyelinated segments (indicated by D). Biopsy of the peroneal nerve contrasted with Osmium tetraoxide, scale bar 50 μm.

**Figure 2:**
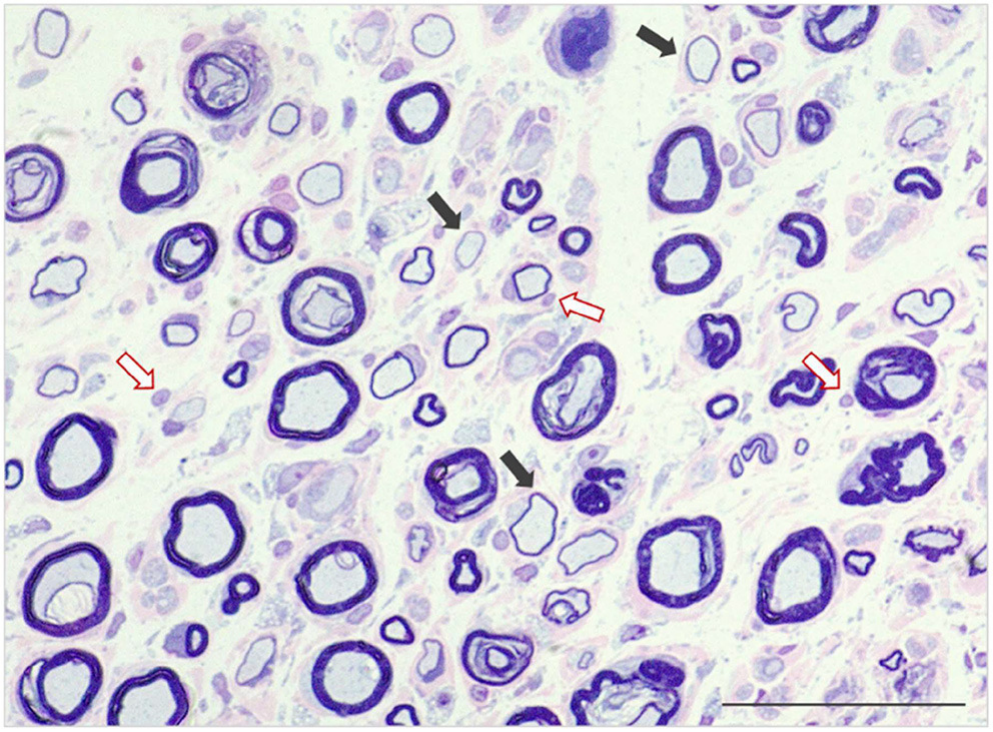
Semithin section of peroneal nerve featuring reduced density of nerve fibers and mild expansion of the endoneurial fibrocollagenous tissue. There are numerous fibers with marked reduction of myelin sheath thickness (demyelination—black arrows) accompanied by multiple fiber adhesive mononuclear cell infiltrates and overall increased endoneurial cellularity (red arrows). Stained with Azurblue Safranin, scale bar 500 μ–m.

Archives of a single European reference laboratory for neuromuscular disorders, MASKED FOR REVIEW, were screened for biopsy diagnosis of inflammatory PN between 2011 and 2019. Only cats with histologic evidence of nerve fiber adhesive and/or invasive inflammatory infiltrates directed at the axons, nodes of Ranvier, and Schwann cells were included (Figures 1, 2). Cats with PN without signs of inflammation in the intramuscular nerve branches and/or peripheral nerve biopsies were excluded from the study. In total 107 cats with inflammatory neuropathy of presumed immune-mediated origin were identified. In all cats peripheral nerve biopsies were available for review and muscle biopsies were submitted in 105/107 cats. Diagnosis was based on findings from main nerve trunk in 105/107 and from intramuscular nerve branches in 2/107. Submitting referral veterinary neurologists were asked to review the medical records of their cases and to contact the cat's owners for follow-up information, before answering the online questionnaire (https://forms.office.com/Pages/ResponsePage.aspx?id=DQSIkWdsW0yxEjajBLZtrQAAAAAAAAAAAAN__iDTpglUQVVXTzNMNlc1UlpFUlo4UEdQOTNOVzRWMy4u). The study was approved by the ethical review board of MASKED FOR REVIEW. The online survey was developed using the online application Microsoft Forms. The questionnaire included 40 single choice, five multiple choice and 48 free text questions. Briefly, five main aspects of medical records were investigated including onset of clinical signs, neurologic examination, findings related to diagnostic work-up, outcome and follow up. Results of electromyography were interpreted and graded by the examiners as follow: minimal (+), mild (++), moderate (+++), and severe (++++) (41).The course of the disease was considered remittent when the cats recovered with none or only minor deficits, and remittent-relapsing if periods of prolonged improvement were followed by relapses. A waxing and waning clinical course implicated persistent clinical signs of variable severity. Recovery was defined as the state in which the cat could walk without assistance and could jump onto objects. Internal review of the questions by the study investigators and external review by clinicians regarding structure, phrasing, understanding, and processing of the questions was performed. All information was obtained directly from the veterinary neurologist who submitted the biopsy. A case questionnaire was considered suitable for enrolments if it was completed to the end and all mandatory questions were answered.

### Statistical Evaluation

Descriptive statistic was performed and data were analyzed by Shapiro-Wilk-test for conformance with a normal distribution. Observation time was defined as the time from the submission of muscle and nerve biopsies to the last contact with the cat owner. Recovery time vs. age was studied using Kruskal-Wallis-test because age did not follow a normal distribution. Recovery time vs. all variables and groups inside of those variables between each other were studied *via* Fisher tests. Disease onset, age, clinical and electrodiagnostic parameters were analyzed for any association with outcome (recovered/not-recovered) via logistic regression. *P* < 0.05 was considered significant. Furthermore, *P*-values were adjusted for multiple comparisons by the Holm method. All analyses were done by the R Statistical Software (version 4.0.3).

## Results

Out of 107 identified candidates, 73 surveys were returned resulting in a response rate of 68.2%. Three were incomplete, thus 70 valid surveys were enrolled for further evaluations. Cats from this survey were geographically distributed among five European countries: Germany (42), United Kingdom (11), Italy (7), France (6), and Switzerland (4).

### Demographics and History

**Figure 3:**
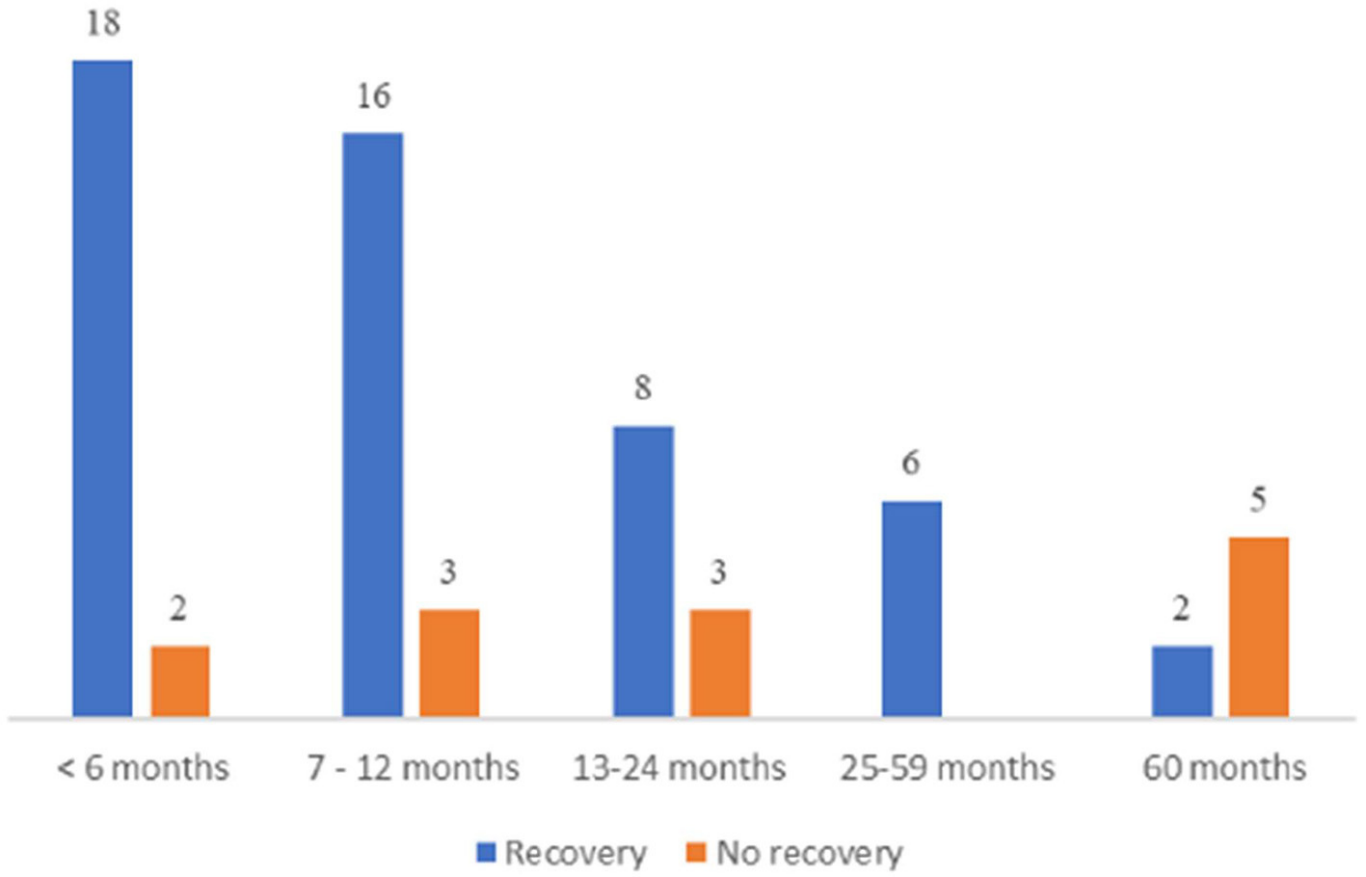
Age at onset. Summary of the outcome in the different age groups with total number of patients for each segment.

The median age at onset of clinical signs was 10 months (range 4–120 months). Muscle/nerve biopsies were collected at a median age of 11 months (range: 4–125 months) (Figure 3). There were 64.3% male (45/70) and 27.1% female (19/70) cats. Sex was not specified in 6 cats. The most common breed was British short hair 25.7% (18/70). Other breeds were domestic short hair 24.3% (17/70), Bengal cat 11.4% (8/70), Maine Coon 8.6% (6/70), Persian cat 5.7% (4/70), mixed breed cat 2.9% (2/70), Thai cat 2.9% (2/70), and one cat each from the following breeds: Ragdoll, Savannah, Siam cat, Siberian cat, Abyssinian cat, unknown breed, Chartreux, Devon Rex, Munchkin, Birman cat, Norwegian Forest cat, Russian Blue, and Scottish Fold. The majority of cats lived indoors (71.2%; 37/52); 7.8% (15/52) had outdoor access. Diet was indicated in 30 cats: 36.7% (11/30) received dry and wet food, 33.3% (10/30) dry food, 20% (6/30) wet food, 6.7% (2/30) biologically appropriate raw food (BARF), and 3.3% (1/30) gluten free diet. In four cats it was reported that other littermates were affected, and in 3 patients, other cats of the same household presented with similar clinical signs according to the owner.

### Preceding Events

Vaccination status was known in 44 cats. Of these, 18.2% (8/44) were vaccinated 6 weeks or less before the onset of clinical signs. There were no reports on tick manifestation in temporal relationship to disease onset. Fifteen cats (21.4%) had a preexisting medical condition or an infection in the 6 weeks prior to disease onset as follows: bite wound (1), gastrointestinal infection (6), signs of feline upper respiratory disease (4), cardiac disease (2), feline leukemia virus (1), patellar luxation (1), arthritis (1), episode with transient ataxia, salivation and mydriasis 18 months before onset of clinical sign (1), and oliguric renal failure (1). In one cat, the symptoms worsened 1 month after neutering. Two cats received medications shortly before onset of signs: acyclovir (one cat) and imidacloprid/moxidectin/praziquantel (one cat).

### Onset and Initial Progressive Phase

Onset was described as acute in 30.9% (21/68) and insidious in 69.1% (47/68) of the cats. No information on onset was available from two cats. An initial progressive phase was described in 74.3% of the cats (52/70). The median duration of the initial progressive phase from onset to peak/plateau of weakness was 14 days (range: 1–180 days). The median time between the onset of weakness and referral for muscle/nerve biopsy was 1 month (range: 0–28 months). The presenting complaints were generalized weakness or tetraparesis in 41 cats; other presenting complaints were pelvic limb weakness (17 cats), inability to jump (13 cats), paraparesis (nine cats), abnormal gait (seven cats), ataxia (four cats), lameness (two cats), unwillingness to play (two cats), paraplegia (two cats), tetraplegia (one cat), ventroflexion of head and neck (one cat), and/or spinal pain (one cat). Additional complaints were gastrointestinal signs in four cats. In 31 cats there was more than one presenting complaint, and in two cats the presenting problem was not specified. Weakness/paresis at the peak/plateau of the disease was characterized as lower motor neuron tetraparesis in 75.7% (53/70), tetraplegia in 2.9% (2/70), paraparesis in 18.6% (13/70) and paraplegia in 2.9% (2/70), respectively. At the peak of the weakness, 47.1% of the cats were non-ambulatory (33/70) and 51.4% (36/70) still able to walk without assistance. In one cat this information was not available.

### Physical/Neurological Examination

**Figure 4:**
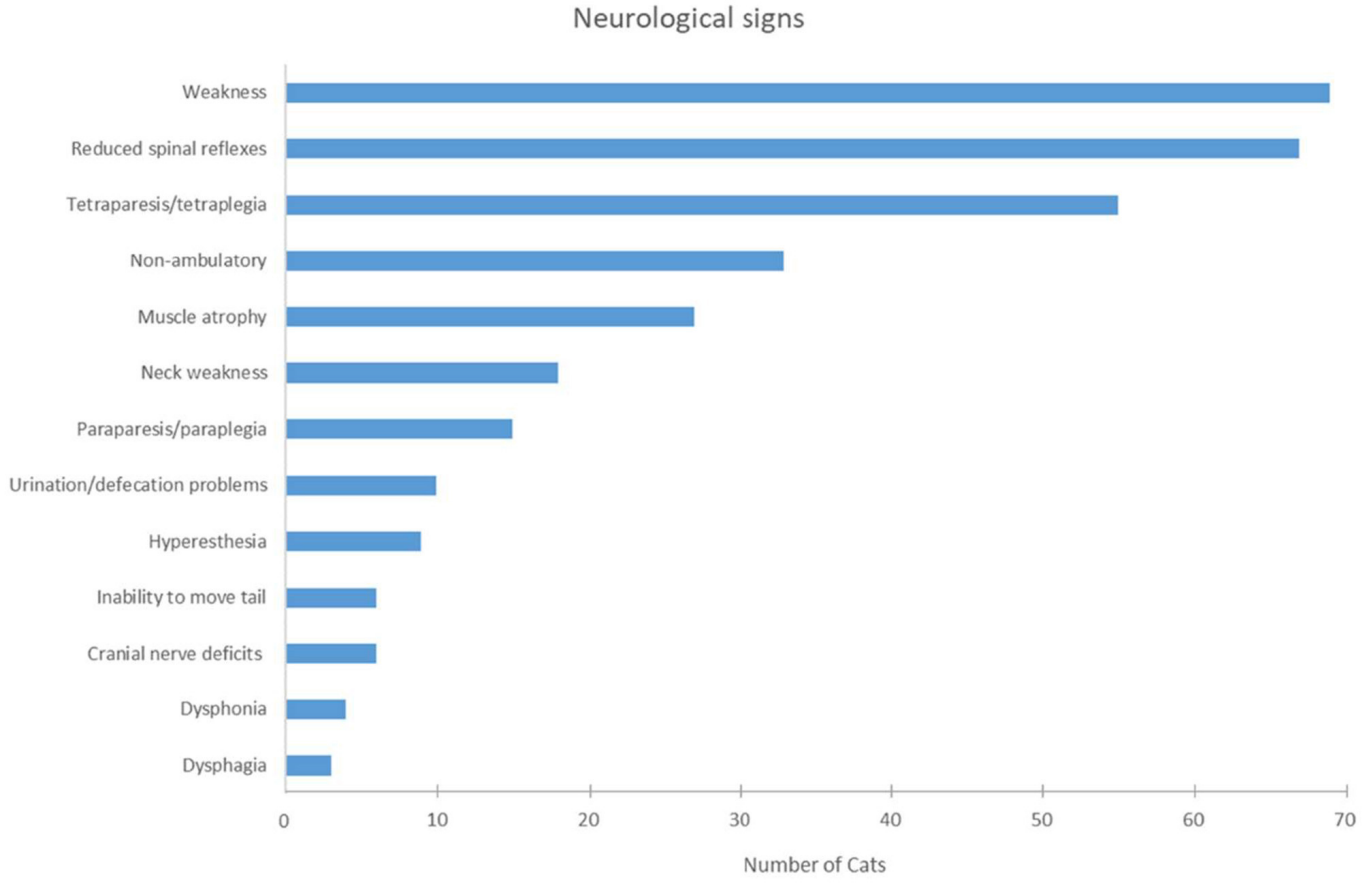
Graphic representation of neurologic signs presented by the cats of this cohort.

Abnormal findings on physical examination were noted in 14 cats as follows: respiratory signs in 5.7% (4/70) (polypnea, increased vesicular lung sounds), small/thin body condition in 2.9% (2/70), heart murmur in 2.9% (2/70) and tachycardia in 1.4%, tail deformity, alopecic tail, bilateral chronic otitis externa, mild generalized lymphadenopathy, pyrexia, and mild ocular discharge in one cat each. Separate questions addressing the presence/absence of weakness, paraparesis/tetraparesis, and the presence/ absence of spinal reflexes were formulated in the survey. Here to follow are the description and summary of the answers collected. Weakness was indicated in all cats but one (98.6%, 69/70) (Figure 4). Paraparesis, inability to move the tail and normal spinal reflexes were described in this cat. In summary, all cats but three had neuromuscular signs on neurological examination (95.7%, 67/70): the withdrawal reflex was reduced in all limbs in 83.6% (56/67) of the cats, in the pelvic limbs only in 13.4% (9/67), and in the thoracic limbs only in one cat, and in one cat affected limbs were not specified. Normal withdrawal reflexes were reported in two cats with paraparesis or tetraparesis, respectively, and reflexes were not described in one cat (tetraparesis). The signs showed a symmetric distribution in 91.4% (64/70) and an asymmetric distribution in 7.1% of the cases (5/70). Inability to move the tail was noted in 8.5% (6/70) of the cats. Neck weakness and flexion of the head and neck was evident in 25.7% of the patients (18/70). Signs of cranial nerve dysfunction were described in 17.1% (12/70) of the cats as follows: dysphonia (four cats), facial paresis/paralysis (four cats), dysphagia (three cats), reduced menace response (one cat), reduced palpebral reflex (one cat). Hyperesthesia was reported in nine cats (12.9%), either on spinal palpation (five cats), limb palpation (three cats), or when lifted up (one cat). Problems with urination or defecation were noted in 14.3% of the cats (10/70). Of these 10 cats, two had defecation problems, two micturition problems, three both, and three were unable to reach the litter or to stand in the litter box. Muscle atrophy was described in 38.6% (27/70) and defined as generalized with greater involvement of the hind limbs.

### Electrodiagnostic Investigations

**Figure 5:**
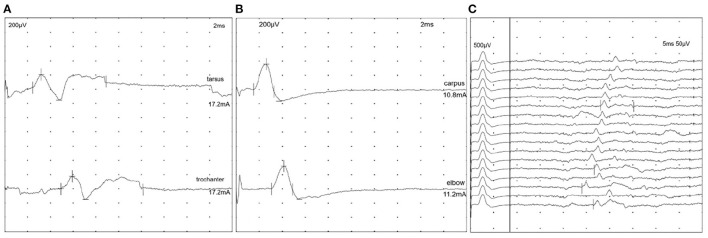
Electrodiagnostic tests of a 14-months-old male Maine Coon cat with presumed immune-mediated polyneuropathy. **(A)** Motor nerve conduction studies of the left sciatic-tibial nerve. CMAPs show a marked reduction of amplitude/area and mild temporal dispersion. Motor nerve conduction velocity is 76 m/s [reference range 93.7 ± 9.4 m/s; Ref. (12)]. **(B)** Motor nerve conduction study of the right ulnar nerve. CMAPs show a marked reduction of amplitude/area. Motor nerve conduction velocity is 64 m/s [reference range 82.1 m/s ± 11.1 m/s; Ref. (12)]. **(C)** F-waves after supramaximal stimulation of the right ulnar nerve at the carpus. F-waves are characterized by a pronounced chronodispersion (variable minimum latency) and a severe increase in latencies. The values of minimum F-waves latencies vary between 24 and 31 ms [reference range 8.4 ± 0.9 ms; Ref. (42)].

Electrodiagnostic studies were performed in 67 cats (67 Electromyography—EMG, 65 Motor Nerve Conduction Velocity—MNCV; 43 repetitive nerve stimulation—RNS; 29 F-waves; 11 Sensory Nerve Conduction Velocity—SNCV; 3 Somatosensory Evoked Potentials—SEP). Review of electrodiagnostic findings was performed considering the following normal reference ranges: MNCV 93.7 +/- 9.4 m/s in the sciatic/tibialis nerve and 82.1 +/- 11.1 m/s in the ulnaris nerve (12). EMG demonstrated spontaneous activity in 89.6% of the cats (60/67). The spontaneous electric activity (SPA) appeared generalized in 93.3% (56/60) and only in the pelvic limbs in 6.7% (4/60); one of the latter cats had only minimal EMG changes in the pelvic limbs. Information on the proximal or distal distribution of EMG changes was available in 74.6% (50/67): in 34% of the cats (17/50), the proximal and distal appendicular muscles were equally affected, in 64% (32/50) the distal muscles and in 2% (1/50) the proximal muscles were more severely affected. Only in one cat the proximal appendicular muscles were more severely affected than the distal appendicular muscles; in this cat the pelvic limbs showed SPA. The predominant abnormalities in EMG were fibrillation potentials in 38.3% (23/60), positive sharp waves in 30% (18/60) and fibrillation potentials together with positive sharp waves in 23.3% (14/60). In five cats EMG abnormalities were not further specified. Only one cat showed additional complex repetitive discharges. Changes were mild in 38.3% (23/60), moderate in 46.7% (28/60) and severe in 11.7% (7/60). EMG changes were considered minimal and appeared only in the pelvic limbs (1/60) or not further described (one cat). Cats that only demonstrated positive sharp waves were more likely to recover than cats with fibrillation potentials (*p* < 0.05). In 65 cats, investigators reported on motor nerve conduction studies (MNCV) (Figure 5). Measured MNCVs (m/s) were available from 43 cats (41 pelvic limb studies, 23 thoracic limb studies), in the others, it was only indicated whether MNCV was decreased or normal. Mean MNCV was 64.6 m/s (14–127 m/s) in the pelvic limbs and 60.8 m/s (15.7–115 m/s) in the thoracic limbs. Taken together MNCV was decreased in 52.3% (34/65) of the cats; specifically, in 49.2% (30/61) of the examined pelvic limbs and in 52.9% (18/34) of the examined thoracic limbs. In cats with a decreased MNCV, MNCV was decreased to 52% in the pelvic limbs and to 65.6% in the thoracic limbs in reference to the lower limit (mean – 2SD) of normal MNCV in cats. Other findings were temporal dispersion in 26.1% (17/65) of the cats which was frequently associated with a decreased amplitude of the CMAP (15/17 cats). A decreased amplitude of the CMAP was a frequent finding (73.8%; 48/65) in general. In total, 81.5% (53/65) of the cats presented with abnormal MNCV, in 17% (11/65) MNCV was normal and in 1.5% (1/65) MNCV was not further specified. Out of the 60 patients with EMG abnormalities, 78.3% (47/60) showed concomitant MNCV changes, 15% (9/60) presented with EMG changes only, and MNCV changes were not indicated in 6.6% (4/60). Repetitive nerve stimulation was performed in 43 cats and was unremarkable in 95.3% (41/43) cats. Cord dorsum potentials were tested in three cats and described as unremarkable in all of them. Further sensory nerve conduction studies were performed in 11 cats and results were unremarkable in all of them. Details about the F-wave evaluation were indicated in 29 cases. In 72.4% (21/29) F-wave was described as abnormal: in 48.3% (14/29) the F-wave was not detected and in 24.1% (7/29) the F-wave was recorded but showed abnormalities described as: increased latency, decreased F-M-ratio, decreased amplitude, inconsistent, and irregular. Only one cat was normal on electrodiagnostic investigation.

### Other Diagnostic Investigations

Advanced diagnostic imaging (Computed Tomography, Magnetic Resonance Imaging of spine/brain) was performed in 20 cats. Abnormal findings were described in four cats: One cat revealed an increased size of the sciatic nerves, one a T2-weighted hyperintensity at the level of L3/L4 (no further details were available), one a diffuse T2-weighted and STIR hyperintensity of several skeletal muscles with contrast enhancement and one a broncho-/pneumopathy and an anomaly of the descending aorta. Results of cerebrospinal fluid analysis (CSF) were available from 26 cats and were unremarkable in 73.1% (19/26). Mononuclear pleocytosis was reported in 3 cats (11.5%; 3/26) and increased protein content (>30 mg/dL) was the only abnormal finding in four other cats (15.4%; 4/26). Acetylcholine receptor antibodies were tested and were unremarkable in nine cats. Increased creatine kinase activity was reported in 51.1% (23/45) and was normal in 48.9% (22/45) of tested cats (reference range: 0–414 U/l). Negative molecular and serologic tests were reported for infectious agents: *Toxoplasma gondii* (42 cats; five cats had a positive IgG titer, but a negative IgM titer), *Feline immunodeficiency virus* (22 cats), *Feline leukemia virus* (22 cats), *Feline coronavirus* (six cats). The following infectious agents were each tested negative in one of the cats: *Bartonella spp, Borna disease virus, Neospora caninum*, and *Encephalitozoon cuniculi*; further details on diagnostic tests were not available for review. Other findings included abdominal lymphadenopathy in two cats, specified as neutrophilic cellular infiltrate by fine needle aspirate in one of these cats, and vertebral spondylosis (Th12-L1), and radiographic evidence of bilateral degenerative joint disease (coxo-femoral joints, stifles) in another cat.

### Treatment

The following treatment modalities were reported: glucocorticoids (70%; 49/70), non-steroidal anti-inflammatory drugs (55.7%; 39/70), L-carnitine supplementation (48.6%; 34/70), and physical therapy/rehabilitation (20%; 14/70). Duration of these therapies was indicated in 20% of cases (14/70) and the median was 8.5 days (range 3–90 days). Other medications included antimicrobial drugs, pyridostigmine and B vitamins. In total, 62.9% of the cats (44/70) were treated before referral for biopsies and 94.3% (66/70) after muscle/nerve biopsies.

### Outcome and Long-Term Clinical Course

Data on outcome and long-term clinical course were available for 63 cats. Of those 63 cats, 10 cats had a follow-up time of <1 month; the median follow-up time in the other cats was 8 months (range: 1–40 months). The overall course of the disease was characterized as remittent in 49.2% (31/63) and remittent-relapsing in 34.9% (22/63) of the cats. Thus, a remittent or remittent-relapsing course was reported in 84.1% (53/63) of the cats. In the others, clinical course was described as stagnant in 7.9% (5/63), progressive in 4.7% (3/63), or waxing-waning in 3.2% (2/63). Cats with a remittent or remittent-relapsing clinical course required a median time of 1.5 months to recover completely or to reach a clinical plateau and stable disease (range: few days to 17 months). Other endpoints were described as follows: the median time within cats could stand without assistance was 7 days, the median time until the cats could walk at least 5 steps or more without assistance was 7 days, and the median time until the cats could jump on objects was 28 days.

**Figure 6:**
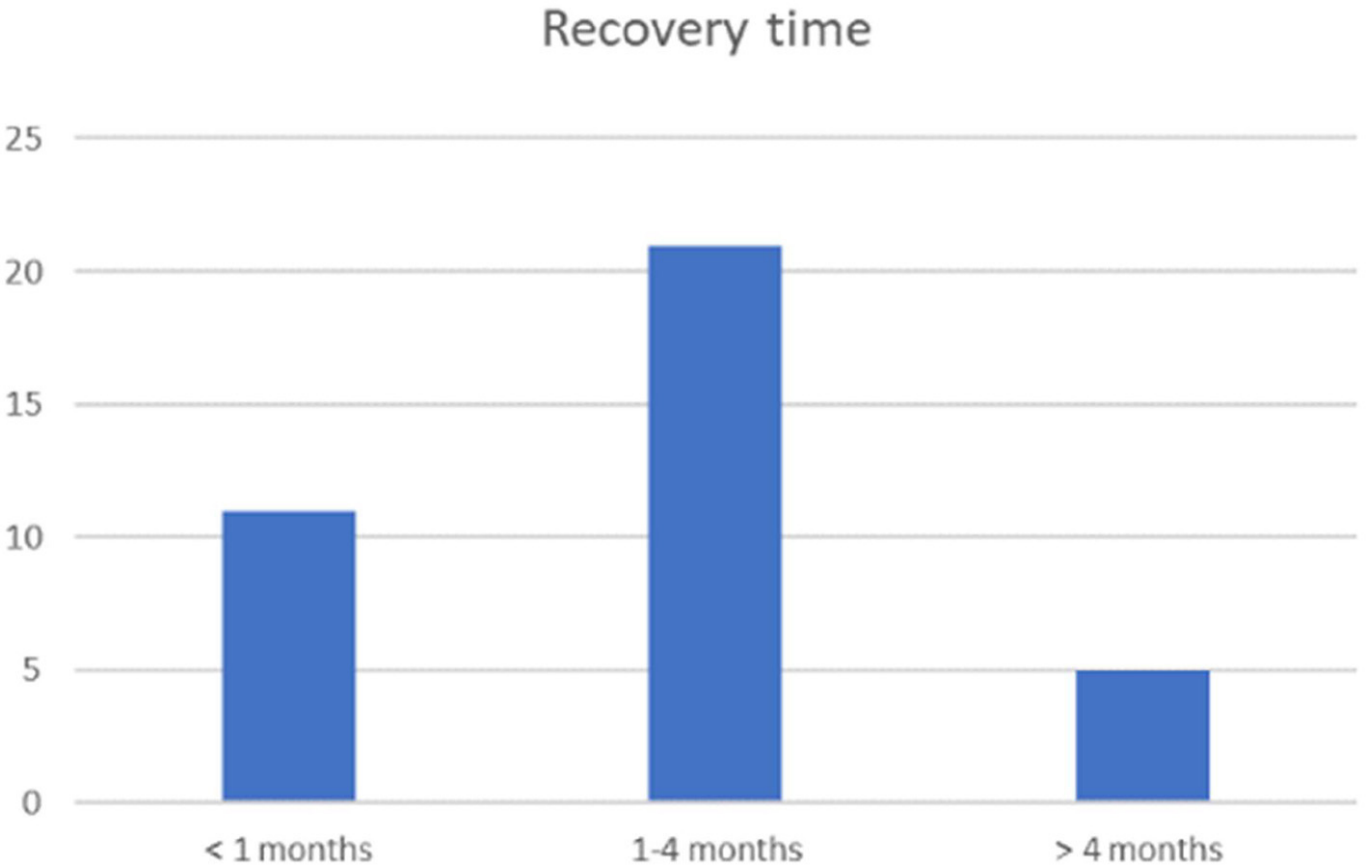
Recovery time. Representation of recovery time in the three groups (weeks: 1–4 months, months: 1–4 months and late: >4 months), with total amount of patients indicated on the y axis.

Relapses were reported in 35.7% (25 cats) (22 remittent-relapsing, two progressive and one waxing-waning course) after a median time of 3 months (range <1–8 months). Cats experienced only one (13/70), two (11/70), or more than three relapses (1/70). In total, 79.4% (50/63) of the cats recovered (Figure 6). Most patients were presented at the plateau of the weakness or during the initial progressive phase. Time from presentation until recovery was available for 37 cats: 29.7% (11/37) recovered within 4 weeks, 56.8% (21/37) within 1–4 months, and in 13.5% (5/37) recovery took longer than 4 months. A younger age at the time of the diagnosis as well as a sudden onset of clinical signs was statistically significant associated with recovery (*p* < 0.05). Only 18.6% of the cats did not recover and 7.4% died or were euthanatized due to PN.

### Correlation Analysis

**Figure 7:**
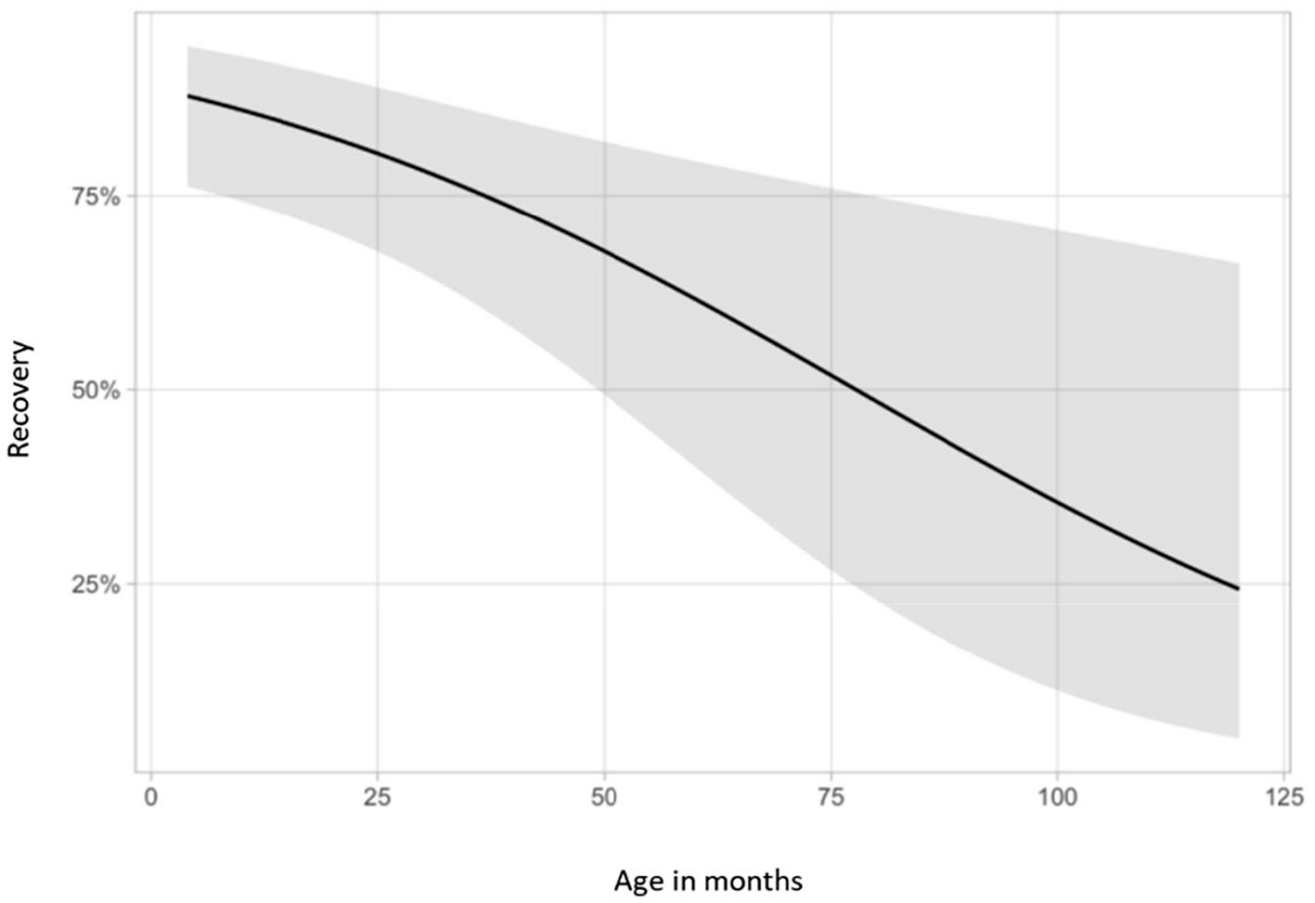
Association between recovery and age at onset. Demonstration of the relationship between recovery and age at onset of clinical signs. The y-axis shows probabilities of recovery predicted by the logistic regression. Younger cats were more likely to recover (*p* = 0.005).

**Figure 8:**
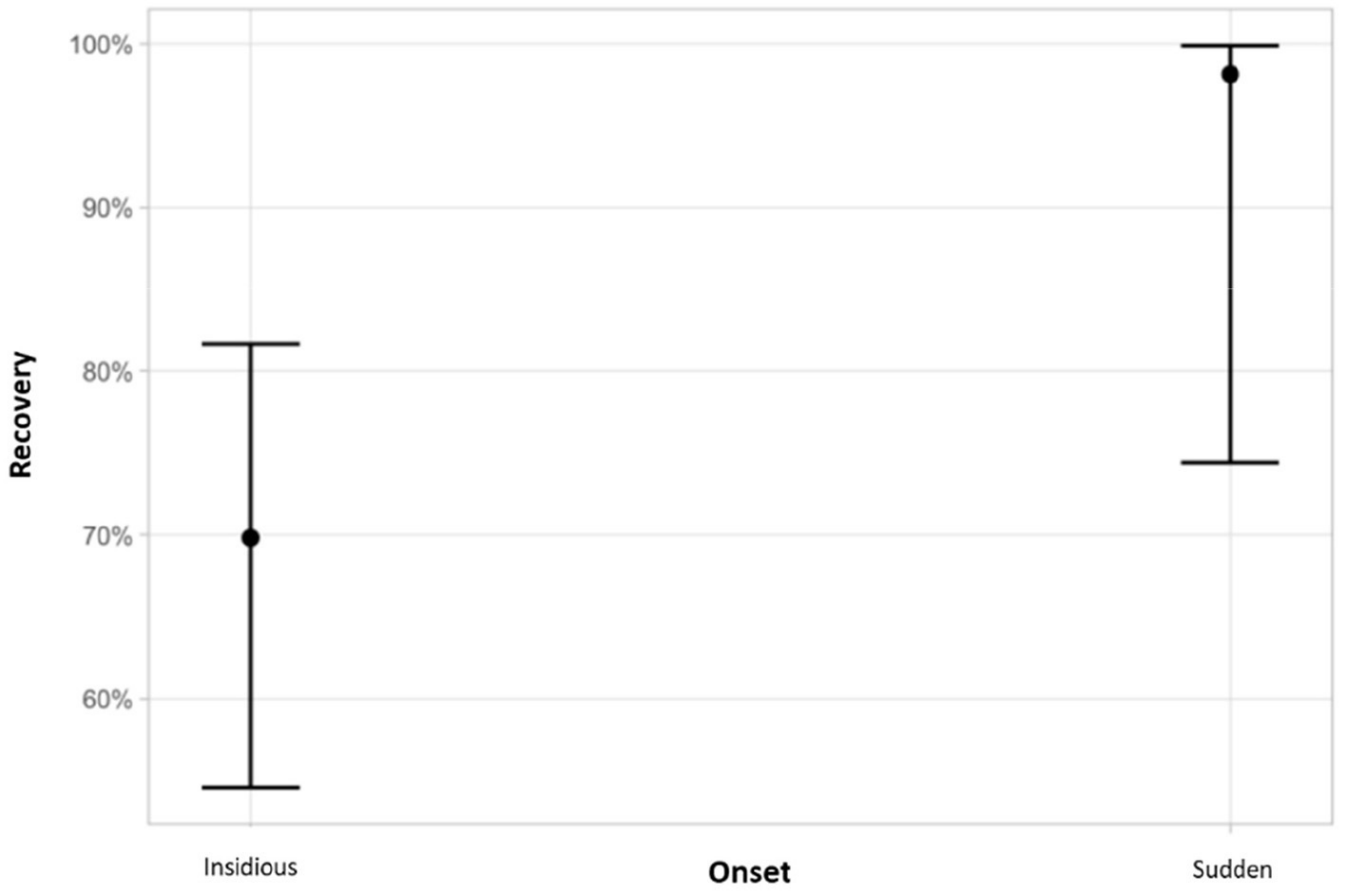
Association between the type of onset and recovery. Cats with a sudden onset of clinical signs were more likely to recover (*p* = 0.037). The y-axis shows probabilities of recovery predicted by the logistic regression. Graph displays estimated probabilities with 95% confidence intervals.

Statistical evaluation of clinical variables and outcome revealed that (1) young age at the time of diagnosis, (2) sudden onset of clinical signs and (3) presence of positive sharp waves alone were significantly associated with recovery (*p* < 0.05) (Figures 7, 8).

## Discussion

This study describes the signalment, clinical sings, electrodiagnostic findings, course, outcome and predictors of outcome of a presumed immune-mediated PN in a large cohort of European cats. Characteristic features were the onset of clinical signs at an early age (median age 10 months), a variety of affected breeds, male predominance, an initial progressive phase that plateaued thereafter, and a remittent or remittent-relapsing course in 84.1% of the cats with overall 79.4% of the cats recovering. Relapses occurred in 35.7% of the cats.

The clinical features of our cohort paralleled previous descriptions from US cohorts: in these, mean age of onset was either described as 10.6 +/- 7.9 months or ranged from 3 to 12 months (31, 35). A variety of breeds were affected in our cohort. The top breeds were British short hair (25.7%) and Domestic short hair cats (24.3%) followed by Bengal cat (11.4%). In previous descriptions, Bengal cats (31, 32, 35) appeared to be predisposed toward development of inflammatory PN (31, 32, 35), but other breeds were also affected (33, 35). British shorthair cats have up to now rarely been associated with an increased risk for immune-mediated PN (43–47). The additional wide representation of domestic short hair cats in our cohort, could suggest a contribution of acquired factors to the inciting cause of inflammatory PN in cats and less of a genetic predisposition than previously assumed.

Possible triggers of immune-mediated events were investigated in the survey, but we were unable to identify unique precipitating factors. Only 18.2% (8/44) of the cats developed clinical signs in the 6 weeks following vaccination. Thus, in most of the cats, the onset had no apparent relationship with vaccination. Concomitant or preceding infection or inflammation of other organ systems was only described in a handful of cats in this study and comprised bilateral chronic otitis externa, mild lymphadenopathy, pyrexia, and ocular discharge among others. Thus, no obvious inciting triggers were identified in this cohort. Nonetheless, a potential contribution of infectious agents in inflammatory PN cannot be excluded as preceding subclinical signs might not be described or detected prior to referral for biopsies. Dietary-related sensory PN from phenylalanine and tyrosine deficiency were described in an experimental group of black cats (29). However, nutritional deficiencies appeared unlikely with regular feeding of high-quality commercial diets and inflammatory changes on peripheral nerve histology. Furthermore, in the history of our cats there was no evidence for exposure to neurotoxic substances such as organophosphates, salinomycin, thallium, mercury, acrylamide, and pyrethrins/pyrethroids or specific medications like vincristine (2, 4, 15–19, 48). Moreover, the course of the disease and widespread geographic origin of the cats throughout Europe would not suggest exposure to poisons either.

Results of the neurologic examination indicated a generalized neuromuscular disorder with a symmetric distribution. Common neurological signs were generalized weakness, tetraparesis and reduced withdrawal reflexes of all limbs, indicative of generalized PN in almost all cats. Less commonly, paraparesis was the presenting feature. Generalized muscle atrophy was a feature in 38.6%. Similarly, previous studies of feline PN reported also clinical signs indicative of generalized neuromuscular disease (1, 2, 5, 31, 32, 35, 37, 39). A proportion of the cats showed additional dysfunction of cranial nerves such as facial paresis/paralysis, swallowing deficits or, dysphonia or autonomic signs like impaired micturition and defecation problems. Other studies from North America and Europe described also decreased palpebral reflex, decreased menace response, decreased gag reflex, and difficulty in prehending food in 21.6–60% (31, 33, 35, 37). Respiratory signs, including polypnea and increased vesicular lung sounds, were reported in 5.7% of the cats but none of the cats developed dreaded consequence of peripheral nerve diseases, such as respiratory paralysis.

The onset of clinical signs was insidious in up to 70% of our cohort (69.1%; 47/68), while acute onset, characteristic of acute polyradiculoneuritis, was reported in 30.9% (21/68) of cats. Interestingly, after onset, an initial progressive phase with deterioration of weakness was described in the majority (74.3%) of the cats. The median time to reach the peak/plateau of weakness was 14 days (mean 23.9 days). An acute onset of generalized neuromuscular weakness with a progressive phase <4 weeks parallels the acute onset of immune-mediated PNs in human medicine and <2 weeks in dogs with acute polyradiculoneuritis. Both, Guillain-Barré syndrome (GBS) and chronic inflammatory demyelinating polyneuropathy (CIDP) can have acute onset in humans but may differ in the subsequent clinical course (49–51). Differences in the onset of clinical signs among different cats might reflect correct observations, but discrepancies could potentially be related to different perceptions of the cats' gait and mobility by cat owners compared to dog owners and humans. Cats do not participate in regular physical activity, as would be the case for dogs, hence precise estimation of the subtle onset of reduced fitness due to neuromuscular disorders might be more challenging to define in feline patients (3). Similar to the observations in our cats, others described the onset of PN in young cats also as either acute or insidious (31, 33, 35).

Electrodiagnostic findings confirmed the presence of generalized neuromuscular dysfunction in nearly all cats. The key finding was the widespread appearance of pathological spontaneous electric activity with a generalized symmetrical distribution together with reduced MNCV. Likewise, previous studies on PN in young cats revealed abnormal spontaneous electrical muscle activity with positive sharp waves and fibrillation potentials accompanied by reduced MNCV, increased latencies and reduced amplitudes (1, 2, 4, 5, 31, 35). Many investigators reported abnormal F-wave studies indicative of nerve root involvement, which is a common finding in acute polyradiculoneuritis in dogs or GBS in humans (49–56). In one cat normal electrodiagnostic results were reported, yet nerve pathology and denervation atrophy of the muscles was graded as moderate. Clinically, this cat showed relatively mild signs (ambulatory tetraparesis), and MNCV was only recorded from one nerve (fibular n.) In this regard it should be highlighted that in human medicine routine electrodiagnostic screening involves comprehensive testing of multiple nerves/muscles which is hardly appreciated in veterinary medicine (57, 58).

A PN with presumptive immune-mediated etiology was indicated by examination of muscle and nerve biopsies that revealed an autoreactive inflammatory neuropathy and failed to provide evidence for an infectious origin in the cats of this cohort. In particular subregion specific immune damage as in nodo-paranodopathy is a quite unequivocal feature of immune-mediated nerve disorder (36). Further support for immune-mediated PN comes from the clinical course which resembles the common PNs in people, GBS and CIDP, in many aspects, with an initial progressive phase until reaching a clinical plateaus/stable disease and subsequent remission in GBS, and chronic or acute onset with remittent-relapsing clinical course in CIDP (49, 50, 59–61). We propose that feline inflammatory PN resembles human CIDP in many aspects. Overall, unequivocal diagnosis of immune-mediated PN can be challenging. In people, diagnosis of GBS and CIDP relies on a combination of neurologic and electrodiagnostic findings (49, 50, 59, 61). Specific challenges are the diagnosis of immune-mediated disease and classification of subtypes. Guidelines in humans for the diagnosis of CIDP are a chronic-progressive course with symmetrical weakness and motor symptoms for at least 8 weeks, non-genetic background and specific electrodiagnostic abnormalities (abnormal distal latency, abnormal MNCV and abnormal F-wave latency) (61). Guidelines for diagnosis of GBS are bilateral flaccid weakness with reduced reflexes, monophasic course with acute onset, elevated protein concentration in CSF examination, specific nerve conduction studies findings (depending on subtype) and exclusion of other causes for weakness (62). Anti-GM2 ganglioside auto-antibody may serve as biomarker and help with definition of subtypes of immune-mediated GBS and CIDP (51, 52), but at present, there are no published data available in cats and sensitivity and specificity appear yet too low for routine use.

Owners may be informed that a high proportion of patients recovers from feline inflammatory PN (79.4%; 50/63). Only 18.6% of our population did not completely recover and 7.4% died from complications or were euthanized due to their PN. Others reported that 12% of the cats were euthanized shortly after diagnosis and 36% of the cats showed only a partial recovery with residual weakness (31) or reported that 3/5 cats were lost on follow up, one cat was euthanized and one cat showed a full recovery (35). In our study cohort, cats with younger age at the time of the diagnosis were more likely to recover as well as cats with a sudden onset of clinical signs (*p* < 0.05). We hypothesize that young cats with polyneuropathy present as a homogenous group with a shared etiology, and that etiology of the polyneuropathy may be different in older cats e.g., related to different immune mechanisms. Cats without fibrillation potentials, that only showed positive sharp waves in the electrodiagnostic studies were more likely to recover (*p* < 0.05). This fact is difficult to explain considering that positive sharp waves and fibrillation potentials reflect the same underlying pathology i.e., denervation. Previous studies had pointed out that there is a discrepancy in the onset of denervation activity with positive sharp waves appearing earlier in the time course of denervation and axonal degeneration than fibrillation potentials (63). We were unable to demonstrate an influence of specific treatment protocols. In human medicine the treatment for GBS is intravenous immunoglobulin therapy or plasma exchange while corticosteroids are ineffective (48–50, 57, 58, 64, 65); on the other hand, use of corticosteroids as well as intravenous immune globulin and plasma exchange proved effective in CIDP patients (66). Most reported treatments in our cohort included glucocorticoids, non-steroidal anti-inflammatory drugs, and nutritional supplementation but clear benefits from their usage could not be identified. Likewise, Bensfield et al. failed to identify an effective treatment protocol due to the retrospective and multicentric nature of their study (31).

Inflammatory PN in young cats showed many similarities to human juvenile CIDP. A male predominance was noticed in the studied population which parallels observations in CIDP (67). The course of the disease was mostly remittent (84.1%) and relapses were a frequent feature occurring in 25 cats (35.7%). These data are in line with previous literature (31, 35). Also in CIDP the characteristic course is remittent-relapsing (59, 61). In the juvenile form of CIDP neurological motor signs predominate, whereas in the adult form sensory deficits are mostly reported (68). Further similarities with our cats are also found in the nerve conduction studies of juvenile CIDP that presents with demyelinating features on MNCV and abnormalities of the F-waves (59).

Based on these results, an inflammatory PN in young cats can be diagnosed from characteristic clinical and electrodiagnostic findings, but nerve biopsies appear still necessary for confirmation in those patients with less clear presentation. An immune-mediated cause appears most likely due to the inflammatory nature of the nerve lesions, the sudden onset or initial progressive phase followed by a plateau, electrodiagnostic findings supportive of demyelinating peripheral nerve disease, and subsequent recovery in most cases. Infectious agents were supported neither by infectious disease testing nor by examination of nerve biopsies. Retrospective multicentric studies present with some limitations and the present one is no exception. As many referral centers and many neurologists were retrospectively involved, there was no standardized way of reporting and measuring results of clinical examination comprising physical and neurological evaluations or electrodiagnostic testing. Besides, collection of data about the outcome was partially affected by missing follow up information in a second opinion referral population.

## Conclusion

We described and characterized an underdiagnosed inflammatory PN with a likely immune-mediated origin in young cats across Europe. Clinical and electrodiagnostic features and the remittent-relapsing clinical course resembled juvenile CIDP in humans in many aspects. The disease in itself has a good prognosis and seems not to be directly influenced by conventional treatments.

## Data Availability Statement

The raw data supporting the conclusions of this article will be made available by the authors, without undue reservation.

## Ethics Statement

The animal study was reviewed and approved by Ethikkommission der Tierärztlichen Fakultät Ludwig-Maximilians-Universität München. Written informed consent was obtained from the owners for the participation of their animals in this study.

## Author Contributions

MR, AF, and JR designed and coordinated the study. NK, MR, and KM provided the data. AF, FW, MR, and KM designed the questionnaire. JR, AF, and MR wrote the manuscript. All authors read and approved the final manuscript.

## Conflict of Interest

The authors declare that the research was conducted in the absence of any commercial or financial relationships that could be construed as a potential conflict of interest.

## Publisher's Note

All claims expressed in this article are solely those of the authors and do not necessarily represent those of their affiliated organizations, or those of the publisher, the editors and the reviewers. Any product that may be evaluated in this article, or claim that may be made by its manufacturer, is not guaranteed or endorsed by the publisher.
